# The Multifaceted Ubiquitination of BIK1 During Plant Immunity in *Arabidopsis thaliana*

**DOI:** 10.3390/ijms252212187

**Published:** 2024-11-13

**Authors:** Junhong Fu, Huihui Wang, Yuling Chen, Chunguang Zhang, Yanmin Zou

**Affiliations:** Ministry of Education Key Laboratory of Molecular and Cellular Biology, Hebei Research Center of the Basic Discipline of Cell Biology, Hebei Collaboration Innovation Center for Cell Signaling and Environmental Adaptation, Hebei Key Laboratory of Molecular and Cellular Biology, College of Life Sciences, Hebei Normal University, Shijiazhuang 050024, China

**Keywords:** BIK1, *Arabidopsis thaliana*, plant immunity

## Abstract

As sessile organisms, the plant immune system plays a vital role in protecting plants from the widespread pathogens in the environment. The *Arabidopsis thaliana* (Arabidopsis) receptor-like cytoplasmic kinase BOTRYTIS-INDUCED KINASE1 (BIK1) acts as a central regulator during plant immunity. As such, not only the BIK1 protein accumulation but also the attenuation is tightly regulated to ensure effective immune responses. Recent studies have highlighted the critical roles of ubiquitination in maintaining BIK1 homeostasis. Here, we review the latest advances in the ubiquitination of BIK1 in plant immunity, which is mediated by ubiquitin ligases PUB25/26, RHA3A/B, RGLG1/2, and PUB4. Additionally, we summarize and discuss the sites and types of BIK1 ubiquitination. Collectively, these analyses not only illustrate that the differential modifications on BIK1 by multiple ubiquitin ligases hold a crucial position in plant immunity but also provide a good example for future studies on ubiquitin-mediated modifications in plants.

## 1. Introduction

As sessile organisms, plants are almost challenged by various microbial pathogens in the environment; therefore, they have evolved an innate immune system to defend against the widespread pathogens. Pattern recognition receptors (PRRs)-mediated immune signaling holds a crucial position in defending against pathogen attacks. PRRs function as the first layer to recognize microbe-/pathogen-derived molecular patterns (MAMPs/PAMPs) or host-derived damage-associated molecular patterns (DAMP) and activate pattern-triggered immunity (PTI) [1,2,3,4,5]. At present, some key PRRs have been identified, such as FLAGELLIN-SENSING2 (FLS2), which perceives the bacterial flagellin (or its derived peptide flg22), EF-TU RECEPTOR (EFR), which recognizes the bacterial elongation factor EF-Tu (or its derived epitope elf18), PEP1 receptor 1 (PEPR1)/PEPR2, which perceive the AtPEPs, and CHITIN ELICITOR RECEPTOR KINASE 1 (CERK1) and LysM-RK LYSINE MOTIF RECEPTOR KINASE5 (LYK5), which recognize fungal cell wall component chitin [6,7,8,9,10]. After binding of PAMPs, PRRs form heterodimeric receptor complexes with their co-receptors, such as BRI1 ASSOCIATED RECEPTOR KINASE 1 (BAK1), and then lead to a series of auto- and trans-phosphorylation events [11,12,13]. The activated receptor complex then phosphorylates receptor-like cytoplasmic kinases (RLCKs), and then the activated RLCKs transmit the signaling to multiple downstream components.

Compared with the typical receptor-like kinases (RLKs), RLCKs only contain a cytoplasmic kinase domain but lack the ectodomain and transmembrane domain. Thereby, most of these RLCKs are localized to the plasma membrane (PM) through N-myristolation or palmitoylation and mainly function in regulating plant cellular activities. In *Arabidopsis thaliana* (Arabidopsis), there are 149 RLCKs, which are divided into 17 subgroups based on the sequence homology [14,15]. Most of these RLCKs play key roles in the regulation of developmental processes, hormone signaling, biotic and abiotic stresses, and so on [16,17,18]. For instance, some of the Arabidopsis RLCK-XII subfamily members regulate BR signaling [15,19,20,21], and many of the RLCK-VII subfamily members are shown to have functions during PRR-mediated immunity, such as PBS1-LIKE (PBL1), PATTERN-TRIGGERED IMMUNITY COMPROMISED RECEPTOR-LIKE CYTOPLASMIC KINASE1 (PCRK1), and PCRK2 [22,23,24,25,26,27].

**Figure 1:**
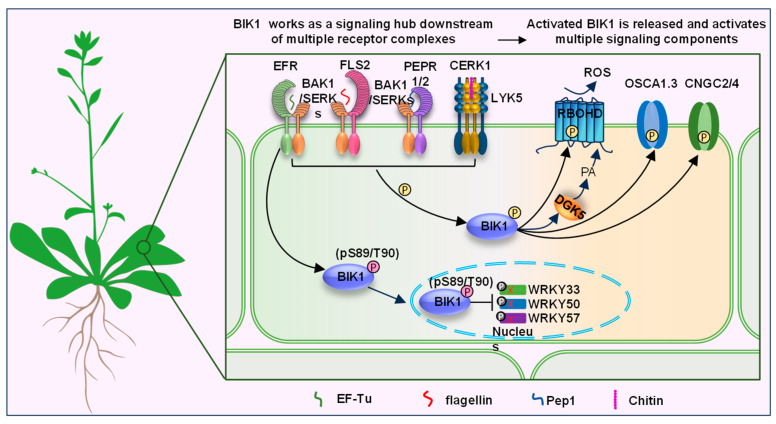
BIK1 acts downstream of multiple PRR complexes and transmits the PAMP/DAMP signaling downstream to induce distinct defenses.

**Figure 2:**
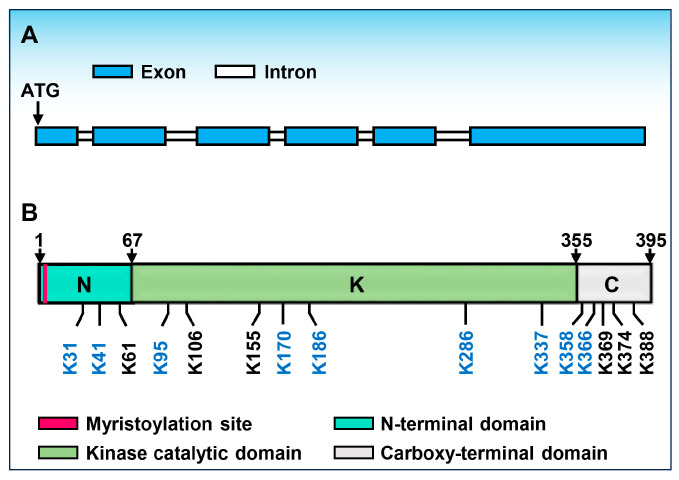
The structures of the *BIK1* gene and BIK1 protein. (**A**). Gene structure of *BIK1*. Blue box indicates exon; white box indicates intron. (**B**). Protein structure and ubiquitylation sites of BIK1. N, N-terminal domain; K, kinase catalytic domain; C, carboxy-terminal domain. Ubiquitinated lysines, which are shown in blue: RHA3A/B ubiquitylation sites.

BOTRYTIS-INDUCED KINASE 1 (BIK1) is one of the RLCK-VII subfamily members [27,28]. In Arabidopsis, BIK1 and other closely RLCKs work as central regulators between multiple receptor complexes and downstream signaling components (Figure 1) [22,23,24,25,26,27,28,29,30,31,32]. The *BIK1* gene is initially identified to be significantly induced during *Botrytis cinerea* (*Botrytis*) infection using an Arabidopsis microarray, thus named *BOTRYTIS-INDUCED KINASE 1* (*BIK1*) [23]. There are six exons and five introns in the BIK1 genomic region (Figure 2A). In agreement with most RLCKs, the coding DNA sequence (CDS) of BIK1 encodes a ser/thr protein kinase, which contains a plant consensus N-myristoylation motif and a kinase catalytic domain (Figure 2B) [23]. In addition, loss of function of the *BIK1* gene leads to severe susceptibility to necrotrophic fungal pathogens *Botrytis* and *Alternaria brassicicola* (*A. brassicicola*), but enhances the resistance to the bacterial pathogen *Pseudomonas syringae pv. tomato* (*Pst*) DC3000, which may be due to the increased salicylic acid (SA) accumulation in the *bik1* mutant [23]. With or without *Botrytis* infection, the level of SA is all higher than wildtype, suggesting that BIK1 acts as a negative regulator of SA accumulation. Moreover, BIK1 is required for normal plant growth and development as well. For example, *bik1* mutant plants exhibit early flowering, reduced fertility, shorter primary roots, longer and more root hairs and lateral roots, and the leaves of *bik1* have serrated margins and wrinkled surfaces compared to wild-type plants, respectively [23]. Subsequently, it is also confirmed that BIK1 functions in ethylene (ET) signaling. Flg22, ACC, and pathogen-induced expression of BIK1 is dependent on EIN3, which is one of the master transcription factors in the ethylene signaling pathway, and *bik1* mutant plants display altered expression of ET-regulated genes [33]. Additionally, BIK1 negatively regulates BR signaling. The *bik1* mutant plants are hypersensitive to brassinosteroids (BR) compared to wild-type plants. Furthermore, upon BR treatment, a leucine-rich repeat (LRR)-receptor kinase BRASSINOSTEROID INSENSITIVE 1 (BRI1), which recognizes the polyhydroxylated growth hormone BR, associates with and phosporylates BIK1, thereby leading to the release of BIK1 from the BRI1 receptor [34]. Recently, BIK1 is reported to interact with and phosphorylate SNF1-related protein kinase 2.6 (SnRK2.6) and controls multiple osmotic stress responses [35].

A series of studies of BIK1 have further demonstrated its important roles in plant immunity. BIK1 protein is required for immune signaling mediated by multiple PRRs, such as FLS2, EFR, PEPR1/2, BAK1, CERK1, and LYK5 (Figure 1) [22,24,25,28,36,37]. Upon PAMPs perception, BIK1 is phosphorylated and released from the PRR complex, leading to the phosphorylation and activation of multiple membrane components, such as the NADPH oxidase RESPIRATORY BURST OXIDASE HOMOLOG D (RbohD), which catalyzes the production of reactive oxygen species (ROS), the Ca^2+^-permeable channel OSCA1.3, two CYCLIC NUCLEOTIDE-GATED CHANNEL (CNGC) proteins, CNGC2 and CNGC4, both of which result in cytosolic calcium influx, and the DIACYLGLYCEROL KINASE 5 (DGK5) which mediate the phosphorylation of diacylglycerol (DAG), leading to phosphatidic acid (PA) burst [22,25,26,28,38,39,40,41,42]. In addition to the plasma membrane (PM) localization through N-myristoylation, BIK1 also localizes to the nucleus to phosphorylate transcription factors WRKY33, WRKY50, and WRKY57, which function in jasmonic acid (JA) and SA regulation, and the phosphorylation of these WRKYs is impaired while BIK1 is phosphorylated at S89/T90 residues by EFR [29]. However, how BIK1 is released from PM and translocated to the nucleus is yet unclear. Furthermore, BIK1 is directly targeted by some bacterial effector proteins, such as the cysteine protease Pseudomonas phaseolicola B (AvrPphB), which cleaves BIK1, and the *Xanthomonas campestris* effector AvrAC, which adds uridine 5′-monophosphate to BIK1 for reducing its kinase activity, also indicating the key roles of BIK1 in immune signaling [28,43]. Therefore, it seems that BIK1 serves as a ‘signaling hub’ to connect the PRR complex and the downstream components, thereby transmitting the PAMP signaling to intracellular signaling and inducing distinct defenses (Figure 1). As such, both the BIK1 protein stability and activation must be tightly regulated to prevent plant autoimmunity and ensure proper responses to pathogens.

Protein ubiquitination is one of the most abundant post-translational modifications in plant immunity. The ubiquitination process involves covalently linking ubiquitins (Ubs) to the target protein through three main reaction sequences: activation, conjugation, and ligation, which are catalyzed by 3 enzymes, respectively: E1 (Ub-activating enzyme, UBA), E2 (Ub-conjugating enzyme, UBC), and E3 (Ub ligase enzyme) [44,45,46]. Firstly, free Ub, which contains 76 amino-acid residues, is activated by the E1 enzyme in an ATP-dependent manner and then transferred from E1 to the catalytic cysteine (cys) of E2. Finally, the E3 cooperates with the E2-Ub conjugate to catalyze the transfer of Ub to the lysine (Lys, K) residues of the substrate proteins [46,47]. Among the 3 enzymes, E3s are the most abundant enzymes in the ubiquitination system and majorly determine the specificity of the substrate for ubiquitination modification. To date, a great deal of studies focus on the importance of E3s involved in plant immunity for avoiding excessive immune responses or regulating the activity of immune components. For instance, during PTI in Arabidopsis, PLANT U-BOX domain-containing E3 ligases PUB12/13 directly polyubiquitinate FLS2 and promote flagellin-induced FLS2 degradation. Moreover, PUB12/13 are phosphorylated by BAK1, which is essential for FLS2-PUB12/13 association, and loss of function of the PUB12/13 genes leads to enhanced immune responses to flagellin treatment [48]. In addition, PUB22, PUB23, and PUB24 are shown to be negative regulators of PTI, and PUB22 mediates the ubiquitination and degradation of Exo70B2, which is one of the exocyst complex subunits [49,50]. Furthermore, PUB22 is phosphorylated and stabilized by the activated MPK3 upon flg22 perception [51]. PBL13 also belongs to the RLCK-VII subfamily; however, it negatively regulates PTI responses through phosphorylating the C terminus of NADPH oxidase RbohD and then leading to the increased polyubiquitination of RbohD, which is catalyzed by PBL13-interacting E3 ligase (PIRE) with and without flg22 perception [52].

**Figure 3:**
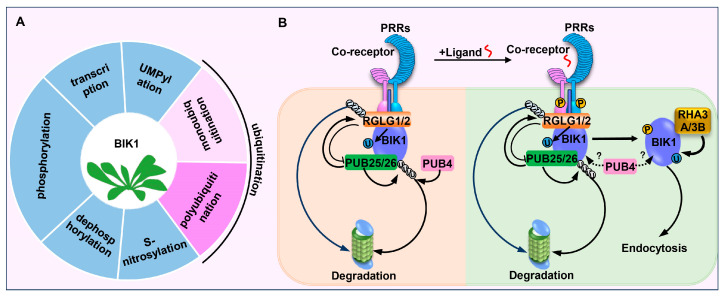
A model of the multifaceted ubiquitination of BIK1 in plant immunity. (**A**) BIK1 is regulated by multiple mechanisms. (**B**) Differential ubiquitination of BIK1 by PUB25/26, RGLG1/2, RHA3A/3B, and PUB4.

BIK1 protein is regulated by multiple mechanisms, such as transcription, phosphorylation, de-phosphorylation, S-nitrosylation, UMPylation, and ubiquitination, or the interplay between them (Figure 3A and Table 1) [33,43,53,54,55,56,57,58,59,60,61,62,63]. Among them, ubiquitination is one of the most abundant types of protein post-translational modification in plant immunity, and multiple studies have demonstrated the key roles of ubiquitination in regulating BIK1 homeostasis. In this review, we aim to summarize and discuss the functions of the differential ubiquitination of BIK1 mediated by seven ubiquitin ligases in PAMP-triggered immune responses. In addition, these advances not only demonstrate that the differential modifications on BIK1 by ubiquitin hold a crucial position in plant immunity but also provide a good example for future research in the insight into the understanding of ubiquitin-mediated modifications in plants.

## 2. Ubiquitin-Mediated Modifications on BIK1

To date, multiple ubiquitin ligases have been identified to ubiquitinate BIK1 since 2018, when the first pair of ubiquitin ligases PLANT U-BOX domain-containing PROTEIN 25 (PUB25) and PUB26 were identified, hence opening a new chapter for the understanding of BIK1 protein stability [56].

### 2.1. PUB25/26 Mediated Polyubiquitination of BIK1

Through a liquid chromatography–tandem mass spectrometry (LC-MS/MS) assay, Wang et al. first identified PUB25 as an interacting protein of BIK1. Then, split-luciferase complementation assays in Nicotiana benthamiana and co-immunoprecipitation (coIP) in Arabidopsis protoplasts further confirmed the association of PUB25 with BIK1. PUB26 is the closest homolog of PUB25, and the subsequent works demonstrated that both PUB25/26 directly interacted with and polyubiquitinated BIK1, which led to the proteasome-mediated degradation of BIK1. In line with this, the BIK1 protein level was greatly reduced and accumulated in the *PUB25-OE* line and *pub25/26* double mutant compared to Col-0 plants, respectively. Furthermore, *pub25/26* mutant plants displayed more resistance to Botrytis and the nonvirulent type III secretion mutant strain *Pst* DC3000 *hrcC^–^*, while *PUB25-OE* transgenic plants showed increased susceptibility to *Botrytis* and *Pst* DC3000 *hrcC^–^* compared to wild-type plants, suggesting that PUB25/26 negatively regulated immunity in Arabidopsis. Interestingly, Wang et al. found that PUB25/26 specifically targeted the hypophosphorylated BIK1 for degradation, thereby preventing excessive immune signaling and maintaining homeostasis of immunity [56]. On the other hand, this also reveals that the interplay between phosphorylation and ubiquitination of BIK1 is essential for BIK1 homeostasis.

Additionally, Wang et al. reported that CALCIUM-DEPENDENT PROTEIN KINASE 28 (CPK28) phosphorylated and activated PUB25/26, while the heterotrimeric G proteins directly inhibited PUB25/26 activity to stabilize BIK1. Thus, Wang et al. demonstrated that PUB25/26, the G proteins, and CPK28 formed a signaling module to regulate the non-activated BIK1 protein accumulation [56]. Moreover, CPK28 is also directly associated with BIK1 in vivo and phosphorylates BIK1 in vitro but does not affect BIK1-PUB25 interaction [56,61]. Liu et al. subsequently found that two closely related RING-finger E3 ubiquitin ligases, ARABIDOPSIS TOXICOS EN LEVADURA31 (ATL31) and ATL6 polyubiquitinated and degraded CPK28, which led to the enhanced accumulation of BIK1 [64]. Bai et al. reported recently that another ubiquitin ligase, RING DOMAIN LIGASE 2 (RGLG2), repressed the ubiquitin ligase activity of PUB25 to maintain BIK1 protein homeostasis [57], collectively suggesting that the regulation of BIK1 homeostasis is more complicated.

### 2.2. RGLG1/2 Mediated Monoubiquitination of BIK1

Apart from regulating the ubiquitin ligase activity of PUB25, RGLG2 also directly ubiquitinates BIK1 [57]. By an RNA sequencing screening, Bai et al. first identified that RING DOMAIN LIGASE 1 (RGLG1), the closest homolog of RGLG2, exhibited a similar expression pattern to BIK1 upon flg22 treatment. Split-luciferase complementation assay in Nicotiana benthamiana indicated the association of RGLG1 with BIK1, which was reduced after flg22 treatment. Subsequently, the authors confirmed that, similar to PUB25/26, both RGLG1 and RGLG2 directly interacted with and ubiquitinated the hypophosphorylated proportion of BIK1. Interestingly, *rglg1/2* mutant plants were more susceptible to *Pst* DC3000 *hrcC^–^* compared to wild-type plants, suggesting that RGLG1/2 positively regulated immune signaling. Further studies showed that RGLG1/2 mediated the monoubiquitination of BIK1 to promote the BIK1 protein accumulation and its association with BAK1, which differed from the actions of PUB25/26 [56,57]. Consistent with this, the overexpression of *RGLG1/2* in Arabidopsis protoplasts resulted in increased accumulation of BIK1, while the BIK1 protein level was reduced in transgenic plants *BIK1 OE/rglg1/2* compared to *BIK1 OE/Col-0*. In addition, the authors also demonstrated that RGLG1/2 competed with PUB25 for binding to BIK1 to maintain an appropriate BIK1 protein level in the resting stage. Upon flg22 perception, the association of RGLG1/2 with either PUB25/26 or BIK1 was reduced, thereby resulting in the degradation of the hypophosphorylated BIK1 by PUB25/26 to prevent further accumulation of activated BIK1. Collectively, Bai et al. demonstrated that RGLG1/2 and PUB25/26 form a module to regulate the homeostasis of hypophosphorylated BIK1. However, it remains unclear how the two pairs of ubiquitin ligases compete for association with BIK1.

### 2.3. RHA3A/3B Mediated Monoubiquitination of BIK1

In addition to RGLG1/2, two transmembrane ubiquitin ligases RING-H2 FINGER A3A (RHA3A) and RHA3B also mediate monoubiquitination of BIK1, but the mechanism is distinct [58]. Given that the disassociation of activated BIK1 from the PRR complex is crucial for subsequent immune responses, the exact mechanism of how to regulate the activated BIK1 remains to be fully elucidated. Ma et al. found that BIK1 was ubiquitinated upon PAMP treatments (flg22, elf18, pep1, and chitin) through an in vivo ubiquitination assay conducted in Arabidopsis protoplasts. The PAMP-induced ubiquitination of BIK1, which was about 8 kDa larger than that of unmodified BIK1, appeared to be monoubiquitination. The authors identified RHA3A as an interacting protein of BIK1 by a yeast two-hybrid screen. RHA3B is the closest homologue of RHA3A. And the subsequent works demonstrated that RHA3A/3B directly interacted with and monoubiquitinated BIK1. Additionally, the *rha3a/b* mutants and the transgenic plants *pBIK1::BIK19KR-HA/bik1*, in which monoubiquitination but not phosphorylation is blocked, exhibited reduced flg22-induced ROS bust and were more susceptible to *Pst* DC3000 *hrcC^−^*, compared to Col-0 and *pBIK1::BIK1-HA/bik1* transgenic plants, respectively. Subsequent studies demonstrated that the monoubiquitination of BIK1 by RHA3A/3B promoted the release of BIK1 from the FLS2-BAK1 complex. This mechanism differs from that of RGLG1/2, which mediate the monoubiquitination of hypophosphorylated BIK1 and promote the BAK1-BIK1 interaction. Hitherto, RGLG1/2 and PUB25/26 target the hypophosphorylated BIK1 for its accumulation and degradation, respectively, while RHA3A/3B target hyperphosphorylated BIK1 for its dissociation from the PRR complex [56,57,58]. Notably, Ma et al. also revealed that the monoubiquitination of BIK1 mediated by RHA3A/3B contributes to its internalization from the plasma membrane to endocytic vesicles for signaling activation, and this flg22-triggered BIK1 endocytosis is distinct from that of FLS2, which is internalized for degradation and signaling attenuation [48,65,66].

### 2.4. PUB4 Mediated Polyubiquitination of BIK1

Besides PUB25/26, there are some other PUBs playing crucial roles in BIK1-mediated immune signaling. For example, two PUB proteins, PUB2 and PUB4, physically associate with FLS2, BIK1, PBL27, and RbohD and enhance BIK1-FLS2 and BIK1-RbohD associations independent of flg22 [59]. PUB4 catalyzes the polyubiquitination of BIK1 as well [60]. However, the regulation of BIK1 homeostasis mediated by PUB4 is more complicated [59,60]. Firstly, Wang et al. found that the *pub2/4* mutant plants exhibited reduced ROS generation, callose deposition, and MAPK activation after PAMP treatment and were more susceptible to the pathogen *Pst* DC3000 *hrcC^−^*, indicating that PUB2/4 play positive roles in the PAMP-triggered immune responses [59,60]. RipAC, which is a type III effector protein from Ralstonia solanacearum, suppresses pattern-triggered immunity. Yu et al. identified PUB4 as an interacting protein of RipAC by a yeast two-hybrid screen, and the subsequent works revealed the positive role of PUB4. For example, *pub4* mutant plants exhibited reduced ROS burst, compromised stomatal closure in response to multiple PAMPs, and displayed more susceptibility to *Pst* DC3000 *hrcC^−^* and *Pst* DC3000 COR^−^, which is a coronatine (COR) deficient mutant of DC3000. However, the MAPK activation was not affected in pub4 mutants, which is different from what Wang et al. demonstrated [59,60]. Notably, the PUB4 protein possesses a U-box domain and six armadillo (ARM) repeats, which are reported to mediate E3 ligase activity and protein–protein interactions, respectively. Intriguingly, Wang et al. unveiled that, although PUB4 was shown to have autoubiquitination activity, the ARM repeats region but not the U-box domain was essential for its function in plant immunity, while Yu et al. discovered that PUB4 mediated the polyubiquitination of non-activated BIK1 for degradation at the resting state. Furthermore, Yu et al. revealed that PUB4 promoted the accumulation of activated BIK1 after PAMP treatment; however, the mechanism is not yet clear [59,60].

## 3. Ubiquitination Sites on BIK1

Typically, ubiquitination of the target proteins occurs on the lysine residues of the substrate proteins to form stable isopeptide (or peptide) linkages with ubiquitin [45,67,68]. There are 30 lysine (K) residues in BIK1 protein, and two residues, K105 and K106, are located in the ATP-binding pocket, which is critical for ATP binding and required for kinase activity [58].

By MS analyses and mutational analyses, Ma et al. and Grubb et al. identified 15 potential ubiquitination sites on BIK1: three in the N-terminal variable domain (K31, K41, K61), seven in the canonical kinase domain (K95, K106, K155, K170, K186, K286, K337), and five in the C-terminal region (K358, K366, K369, K374, K388) [58,69] (Figure 2B). Among them, nine ubiquitination residues of BIK1 (K31, K41, K95, K170, K186, K286, K337, K358, K366) are targeted by RHA3A/B (Figure 2B). Ma et al. demonstrated that individual lysine mutations did not affect monoubiquitination of BIK1 by mutational analyses in vivo, thus the monoubiquitination may not be restricted to a single lysine; once a single site is mutated, the alternative sites could still be ubiquitylated [31,58]. Moreover, the mutant variant BIK1(9KR) largely blocks flg22-induced BIK1 monoubiquitination and the dissociation from the PRR complex. Apart from the BIK1(9KR), the mutant variant of the N-terminal five lysine residues (BIK1(N5KR)) or C-terminal four lysine residues (BIK1(C4KR)) partially affects the BIK1 ubiquitination [58]. However, RGLG1/2 still promotes the protein accumulation of BIK1C4KR, and PUB25 also mediates the degradation of BIK1C4KR, suggesting that the C-terminal ubiquitination sites of BIK1 mediated by RHA3A/B may not be targeted by RGLG1/2 and PUB25/26 [57].

Notably, the materials used for MS analyses in the studies of Ma et al. and Grubb et al. were treated with PAMP, flg22, or elf18, respectively [58,69]. However, RGLGs and PUBs target the non-activated proportion of BIK1, so it is possible that there are some new ubiquitination sites of BIK1 that have not been discovered. Therefore, it is important to develop new methods to identify the respective sites of BIK1 mediated by RGLGs and PUBs, thereby elucidating how these E3 ligases synergistically regulate the protein stability of BIK1.

## 4. Ubiquitination Types

With respect to the ubiquitination types, there are monoubiquitination and polyubiquitination with at least eight different linkages [45,47]. Monoubiquitination is formed by the transfer of a single Ub to the lysine residue of a substrate protein. When multiple lysine residues become modified with one ubiquitin, leading to multimonoubiquitylation. Furthermore, if ubiquitination occurs on lysine residues of a ubiquitin, which is already attached to the substrate protein, leading to the formation of polymeric chains. Moreover, the C-terminus of Ub can be attached to any of the seven internal lysine residues (K6, K11, K27, K29, K33, K48, and K63) or the N-terminal methionine (Met1) of ubiquitins that are already substrate-bound, thereby leading to at least eight different linkages. During the Ub elongation, if the same lysine residue of Ub is modified, the formed polyubiquitin chains are homogenous, as in the K48- or K63-linked chains. If different residues link alternately, the chains are heterotypic, mixed, and branched polyubiquitin chains, as in the K63/Met-linked chain [45,47,67,70,71,72,73,74,75]. Different types of ubiquitin modification may result in distinct fates for the substrate proteins [45,76]. Of the various modes of ubiquitination, K48- and K63-linked polyubiquitination are the best-known types. Proteins marked by K48-linked polyubiquitination are always targeted to the 26S proteasome for degradation, while monoubiquitination, K63-, or Met1-linked ubiquitination usually leads to a non-degradative fate for regulating the substrate’s activity, localization, protein trafficking, DNA repair, endocytosis, and so on [76,77].

The types of BIK1 ubiquitination mediated by these ubiquitin ligases are different. For example, PUB25/26 and PUB4 trigger the polyubiquitination of BIK1 [56,60]. RGLG1/2 and RHA3A/B mediate the monoubiquitination of BIK1 [57,58]. Recent advances revealed that RGLG1/2 and PUB25/26 could catalyze multiple types of ubiquitin modification. For instance, RGLG1/2 is shown to catalyze K48, K63-linked polyubiquitination, and monoubiquitination [57,78,79]. K63-linked ubiquitination has been shown to regulate the internalization and endocytotic trafficking of membrane-localized receptors. As reported, RGLG1/2 could catalyze K63-linked polyubiquitylation of the auxin transport protein PIN-FORMED 2 (PIN2) to modulate apical dominance and function in endosomal sorting decisions [78,79]. Notably, RGLG1/2 also mediates the degradation of MAPKKK18 and transcription factor ERF53 to control drought responses likely via K48-linked polyubiquitylation [80,81]. Moreover, RGLG2 could move to the nucleus from the plasma membrane under drought stress; however, the mechanism is not yet clear. RGLG1 and RGLG5 are found to ubiquitinate PP2CA, a negative regulator of abscisic acid (ABA) signaling, and lead to proteasome-mediated degradation.

PUB25/26 have been reported recently that they could catalyze both K48- and K63-linked polyubiquitination as well. For example, PUB25/26 interact and mediate the polyubiquitination of the transcriptional regulator INDUCER OF C-REPEAT BINDING FACTOR EXPRESSION1 (ICE1) and MYB15 via K48-linked polyubiquitylation for protein degradation, while catalyzing K63-linked polyubiquitylation of them to enhance their stability, thus dynamically modulating the stability of ICE1 and MYB15 at different stages of the cold response [82]. However, does PUB25 or PUB26 mediate both K48- and K63-linked polyubiquitination of BIK1 in plant immunity? And how do RGLG1/2 or PUB25/26 catalyze the ubiquitination of multiple substrates with different ubiquitin modifications? Although E3 ubiquitin ligases hold key roles in the specificity of substrate recognition and subsequent protein fate, the ubiquitin conjugate E2-E3 pairs mainly determine the types of ubiquitination [45,76]. As reported, RGLG2 has been shown to interact with E2 ubiquitin-conjugating enzyme UBC35, a plant homolog of the yeast ubiquitin conjugation enzyme UBC13, which is known to be capable of catalyzing the formation of K63-linked ubiquitin chains [79,83]. Thus, it will be interesting to identify which E2 interacts with the seven ubiquitin ligases for mediating the BIK1 ubiquitination and whether the E2s are the same or different ones to interact with distinct E3s of BIK1.

## 5. Conclusions and Perspectives

Taken together, this study reviews the multifaceted regulations of BIK1 homeostasis by ubiquitination in plant immunity. BIK1 serves as an essential component of plant immunity; as such, not only the BIK1 protein accumulation but also the attenuation is tightly regulated to ensure proper immunity. The present studies altogether suggest that PUB25/26, RHA3A/3B, PUB4, and RGLG1/2 form a regulatory network to regulate BIK1 homeostasis via different ubiquitination types (Figure 3B). In the resting stage, BIK1 is monoubiquitinated by RGLG1/2 and polyubiquitinated by PUB25/26 and PUB4 to dynamically maintain the BIK1 protein level. RGLG1/2 and PUB25 negatively regulate each other and antagonistically control the accumulation of non-activated BIK1 protein. And PUB4 targets and degrades non-activated BIK1 as well. Upon flg22 treatment, BIK1 is activated by the PRR complex and then monoubiquitinated by RHA3A/3B, leading to the disassociation of activated BIK1 from the PRR complex, ultimately resulting in the activation of multiple downstream components. Meanwhile, flg22 also induces the disassociation of PUB25/26 with RGLG1/2 and the phophorylation of PUB25/26 by CPK28, both of which enhance the E3 ligase activity of PUB25/26. This leads to the degradation of the hypophosphorylated portion of BIK1, indirectly limiting the pool of the activated BIK1 pool and preventing excessive immune activation. Moreover, PUB4 could enhance the accumulation of activated BIK1 following PAMP treatment with an unclear mechanism.

Despite these breakthroughs in the understanding of BIK1 ubiquitination, many important questions remain unclear. For example, (1) since CPK28-mediated phosphorylation positively regulates the activity of PUB25/26, is the activity of RGLGs or RHA3A/3B regulated by the phosphorylation of kinases? Such as CPK28, BIK1, or other kinases? (2) How do these ubiquitin ligases function orchestrally to maintain BIK1 homeostasis? (3) Given that seven ubiquitin ligases regulate the homeostasis of BIK1, what is the biological significance of this kind of regulation? Furthermore, do these regulations differ at different tissues, distinct developmental stages, or under various stress conditions? (4) Considering that the monoubiquitination of activated BIK1 mediated by RHA3A/3B plays a role in endocytosis, what are the exact outcomes of RGLG1/2-mediated BIK1 monoubiquitination? Are there other E3 ubiquitin ligases that target and degrade hyperphosphorylated BIK1? What is the ultimate fate of hyperphosphorylated BIK1? (5) How are the orthologs of AtBIK1 regulated in other crops, and do they also undergo regulation by multiple ubiquitin ligases? Answers to these questions will shed new light on our understanding of ubiquitination in plant immunity.

## Figures and Tables

**Table 1:** The post-translational modifications of BIK1.

Post-Translational Modification	Responsible Regulators	Molecular Function	Function	Reference
Ubiquitination	PUB25/26	Hypophosphorylated BIK1 degradation	Immune signalling	[56]
	RGLG1/2	Hypophosphorylated BIK1 accumulation	Immune signalling	[57]
	RHA3A/3B	BIK1 release; BIK1 endocytosis	Immune signalling	[58]
	PUB4	Hypophosphorylated BIK1 degradation; activated BIK1 accumulation	Immune signalling	[59,60]
Phosphorylation	BAK1	BIK1 activation and stabilization	Immune signalling	[22]
	EFR	BIK1 activation	Immune signalling	[29]
	PEPR1/2	BIK1 activation	Immune signalling	[4,5,24]
	CERK1/LYK5	BIK1 activation	Immune signalling	[9,10]
	BRI1	BIK1 activation	BR signaling	[34]
	CPK28	Negative regulator of BIK1	Immune signalling	[61]
	MAP4K3/4	BIK1 stability	Immune signalling	[53,55]
De-phosphorylation	PP2C38	Negative regulator of BIK1	Immune signalling	[62]
S-nitrosylation		BIK1 activation and stabilization	Immune signalling	[54]
UMPylation	AvrAC	Reducing BIK1 kinase activity	Immune signalling	[43]
